# Development and validation of a predictive model for immune-related genes in patients with tongue squamous cell carcinoma

**DOI:** 10.1515/biol-2022-0469

**Published:** 2022-12-15

**Authors:** Meng Yang, Changyu Zeng, Zhongcheng Gong, Bo Shao, Gaocheng Liu, Xuying Bao, Bin Nie

**Affiliations:** Department of Stomatology, Urumqi Stomatological Hospital, No. 196 Zhongshan Road, Tianshan District, Xinjiang Uygur Autonomous Region, Urumqi, China; Center for STD/AIDS Control and Prevention, Xinjiang Uygur Autonomous Regional Center for Disease Control and Prevention, Xinjiang Uygur Autonomous Region, Urumqi, China; The Oncological Department of Oral and Maxillofacial Surgery, The First Affiliated Hospital of Xinjiang Medical University, Xinjiang Uygur Autonomous Region, Urumqi, China

**Keywords:** tumor-associated immune, immune infiltrates, prognosis, tongue squamous cell carcinoma

## Abstract

The present study involved building a model of immune-related genes (IRGs) that can predict the survival outcomes of tongue squamous cell carcinoma (TSCC). Using the TCGA database, we collected the gene expression profiles of patients with TSCC and analyzed the differences in IRGs obtained from the ImmPort database. Subsequently, we constructed a predictive model. Transcription factors and differentially expressed IRGs can be used to construct TSCC regulatory network. CIBERSORT tool was used to analyze the relative proportion of 22 tumor-infiltrating immune cells in TSCC samples. Finally, a prognostic model is constructed. We established an IRG model formed by seven genes. The receiver operating characteristic value of the prognostic model based on IRGs is 0.739. After the analysis of the correlation between IRGs and clinical and pathological conditions, we found that Gast was related to grade, IRF9, LTB, and T stage. Among the 22 tumor-infiltrating immune cells, the resting natural killer (NK) cells were found to be related to the 5-year survival rate. This study constructed a prognostic model formed by seven IRGs and discussed the tumor-infiltrating immune cells, which are related to the survival outcome, reflecting the potential regulatory role of TSCC tumor immune microenvironment that could potentially promote individualized treatment.

## 1 Introduction

Tongue squamous cell carcinoma (TSCC) has the highest incidence among head and neck squamous cell carcinomas, accounting for one-third of all oral cancers worldwide [1]. It shows high growth rate and transfer potential [2,3]. According to 2019 statistics [4], the estimated death toll due to TSCC was predicted to be 3,020 in America, which had increased by 182.2% compared to the death toll of 1,070 in 2006 [1]. TSCC has a better prognosis in its early stage, with a death rate of only 19% [5]; however, the 5-year survival rate of patients with advanced disease is only 40–60%, which has hardly improved in the past 20 years [6]. The development of new and sensitive biomarkers to improve the monitoring of this disease has helped to improve the clinical outcomes of patients with TSCC.

Recently, the tumor microenvironment has raised serious concerns. The tumor microenvironment represents the dynamic state and complicated interaction between the matrix and cancer cells. The malignant phenotype of the tumor is not only determined by the intrinsic activity of the tumor cells, but also by the immune cells recruited and activated by the tumor cells. Ehrlich was the first to propose the immunological surveillance concept, considering tumor cells to emerge in the body that can be recognized and eliminated as foreign matter by the immune system [7]. The function and composition of tumor-infiltrating immunocytes change subtly based on the parasite’s host immune state, and they have been used as valid targets of therapeutic drugs [8,9]. The inhibitory effects of immune-related genes (IRGs) have attracted much interest. A study conducted by Shau-Hsuan Li et al. [10] showed that suppressing the activity of the mammalian target of rapamycin in TSCC cell lines and a mouse model results in benign tumor inhibition. Wu et al. [11] demonstrated that human telomerase reverse transcriptase knockout attenuates nuclear factor-κB signaling via negative feedback regulation during tumor progression. Weng et al. [12] found that microRNA (miR)-373-3p activates Wnt/β-catenin signaling by directly targeting the dickkopf WNT signaling pathway inhibitor 1, thereby promoting TSCC transfer induced by epithelial–mesenchymal transition (EMT), suggesting that miR-373-3p can be a potential target for TSCC therapy. These findings provide a new strategy for immune-based treatment of cancer.

The above-mentioned findings demonstrate the significance of understanding the immunological mechanisms of TSCC. The availability of massive gene expression datasets from public databases provides necessary impetus for immunotherapy research. However, studies using IRGs for the co-construction on prognostic models and correlation studies on the function of tumor-infiltrating immunocytes in TSCC are rare. To the best of our knowledge, no models based on IRGs can be used for the diagnosis, treatment, and prognosis of TSCC. Transcription factors (TFs) are important molecules that directly control the degree of gene expression. Hence, it is necessary to probe the potential of TFs to adjust these clinically relevant processes. We used the data available on The Cancer Genome Atlas (TCGA) database to create an immune-related risk model made up of seven IRGs by analyzing their differential expression in a prognostic landscape and integrating clinical and pathological indexes to perform correlation tests. We expect this study to be a foundation for follow-up research on immune-based strategies to facilitate the personalized treatment of patients with TSCC and improve their clinical outcomes.

## 2 Methods

### 2.1 Clinical samples and original data acquisition

Approximately 162 gene expression profiles of TSCC samples and relevant clinical information were downloaded from TCGA data portal (https://cancergenome.nih.gov/). A total of 147 tumor samples and 15 normal samples were used for further analyses. A list of IRGs was obtained from the ImmPort (http://www.immport.org) database.

### 2.2 Differential gene analysis

The analysis of differential gene expression in the tumor and non-tumor samples from the transcriptome RNA-sequencing data was accomplished using *R* language, data processing by Wilcoxon test, intake agreeable data, setting a false discovery rate <0.05, and a log2|fold change| >1 as the cutoff value. Subsequently, we extracted IRGs from these differentially expressed genes. We then performed the Kyoto Encyclopedia of Genes and Genomes (KEGG) enrichment analysis to study the differential expression of IRGs.

### 2.3 Construction of a regulatory network of TFs with IRGs

TF genes were obtained from the Cistrome Cancer database, which contains 318 TFs and provides regulatory links between TFs and transcripts [13]. We extracted the TFs pertinent to IRGs that showed differential expression and used the Cytoscape software version 3.6.1 to show the potential network relation [14].

### 2.4 Prognostic model based on IRGs

Using univariate Cox regression analysis, we selected IRGs that showed a significant correlation with the overall survival (OS) of patients with TSCC. Subsequently, these survival-related genes were subjected to multivariate Cox regression analysis, and IRGs independently related to the prognosis were used to construct a prognostic model. A linear combination of the relative gene expression levels multiplied by regression coefficients constitutes the prognostic model; the coefficients in this model represent the relative weights of genes in multivariate cox regression analysis. Using the median value of the prognostic model as the risk cutoff value, we divided the patients into high- and low-risk groups and compared the survival outcomes of the two groups. The survival curve was plotted using the Kaplan–Meier (K–M) method. We then used *R* to plot the risk curve according to the patient’s risk value.

Univariate and multivariate Cox regression analyses were performed for IRGPM, sex, grade, stage, and histological grade. IRGs and clinical parameters involved in model construction were merged with Perl language, and independent *t*-tests were used to analyze the correlations between IRGs and clinical and pathological indicators, such as age, sex, stage, grade, and T and N in the prognostic model.

### 2.5 Real-time quantitative reverse transcription-polymerase chain reaction (qRT-PCR)

qRT-PCR was used to determine the gene expression levels in the tissues of patients with TSCC and healthy controls. According to the manufacturer’s instructions, SuperScriptTM III reverse transcriptase (Invitrogen) was used to reverse transcribe the total RNA to cDNA. Gene expression was analyzed using qPCR SYBR Green Master Mix (CloudSeq, Shanghai) on a QuantStudio5 real-time PCR system (Thermo Fisher).

### 2.6 Evaluation of tumor-infiltrating immune cells

CIBERSORT (https://cibersort.stanford.edu) software was used to analyze the relative levels of 22 tumor-infiltrating immune cells in a complex gene mixture [15]. Transcriptome data were transformed using the R language “limma” software package to obtain a standard input data file for CIBERSORT software. Another set of input files consisted of a matrix file of 547 genes, containing the gene expression values. CIBERSORT software used the gene expression deconvolution algorithm to calculate the relative proportion of 22 tumor-infiltrating immune cells in each sample. The relationship between the 22 tumor-infiltrating immune cells and the 5-year survival rate was calculated using the R language “survival” package. Data with *P* < 0.05 were used for further analysis.

### 2.7 Statistical analysis

We used the Perl software (version 5.30) to perform the original data reduction and Wilcoxon test to conduct the variation analysis. Univariate cox regression analysis was used to assess the correlation between OS and IRGs, while multivariate cox regression analysis was used to construct the prognostic model. The receiver operating characteristic (ROC) survival curve was plotted using the R language “survival” package. The area under the curve was used to verify the accuracy of the model [16], and an independent sample *t*-test was used to examine the relationship between the IRGs involved in model construction and clinical parameters, such as the pathological stage. Statistical significance was set at *P* < 0.05.

## 3 Results

### 3.1 Differentially expressed IRGs

**Figure 1:**
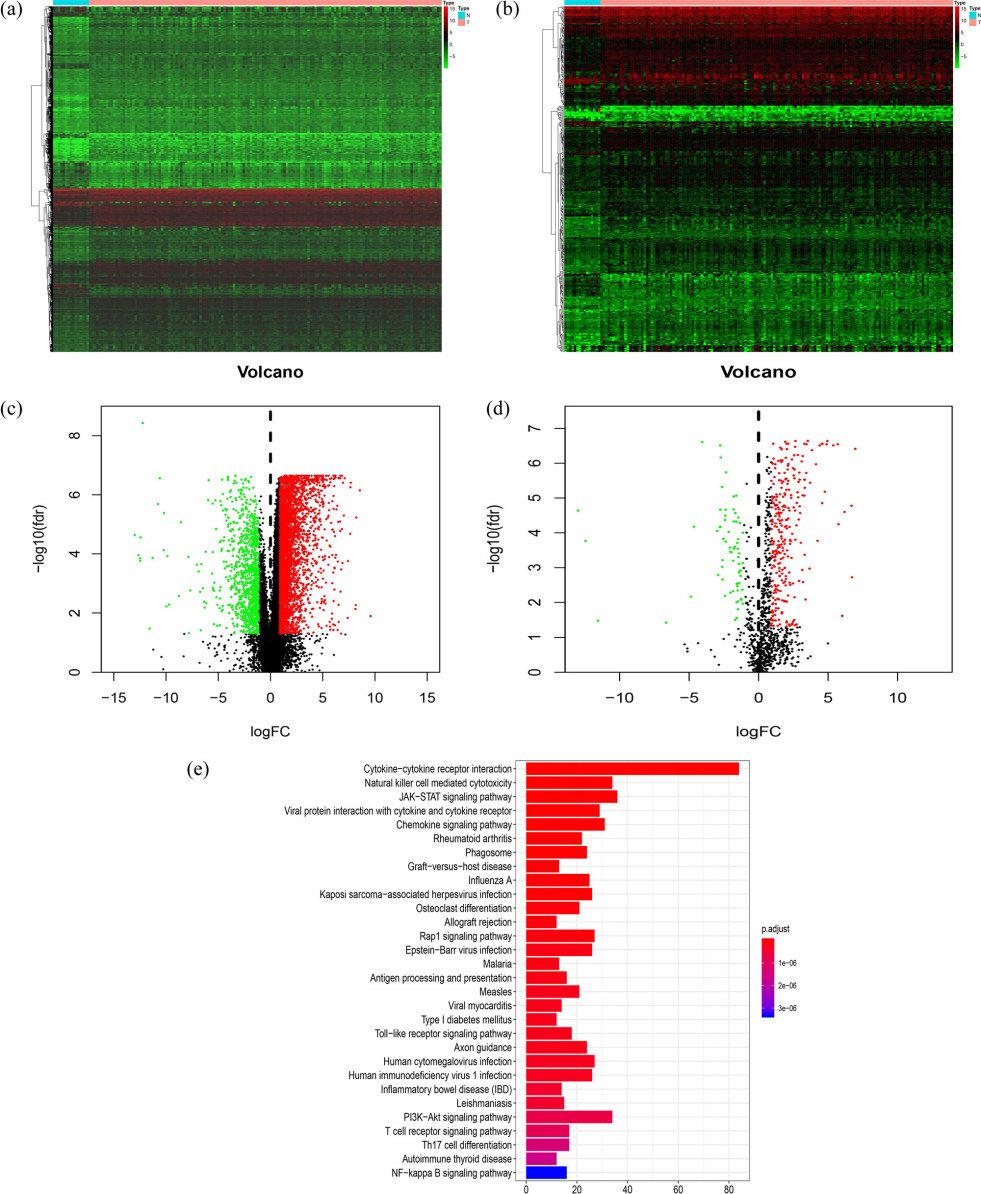
Differentially expressed IRGs: heatmap (a) and a volcano plot (c) demonstrate the differentially expressed genes between TSCC and non-tumor tissues; red dots represent differentially expressed genes and black dots represent non-differentially expressed genes. Differentially expressed IRGs are shown in the heatmap (b) and a volcano plot (d); red dots represent differentially expressed genes and black dots represent non-differentially expressed genes. (e) Functional enrichment analysis using KEGG revealed that these genes were actively involved in a cytokine–cytokine receptor interaction pathway. TSCC, tongue squamous cell carcinoma; IRGs, immune-related genes; KEGG, Kyoto encyclopedia of genes and genomes.

From the 3,692 upregulated and 995 downregulated genes that were identified (Figure 1a and c), we screened 287 upregulated and 68 downregulated IRGs (Figure 1b and d). Figure 1e summarizes the results of KEGG enrichment analysis, showing that the cytokine–cytokine receptor interactions constituted the most common pathway.

### 3.2 TF regulatory network

**Figure 2:**
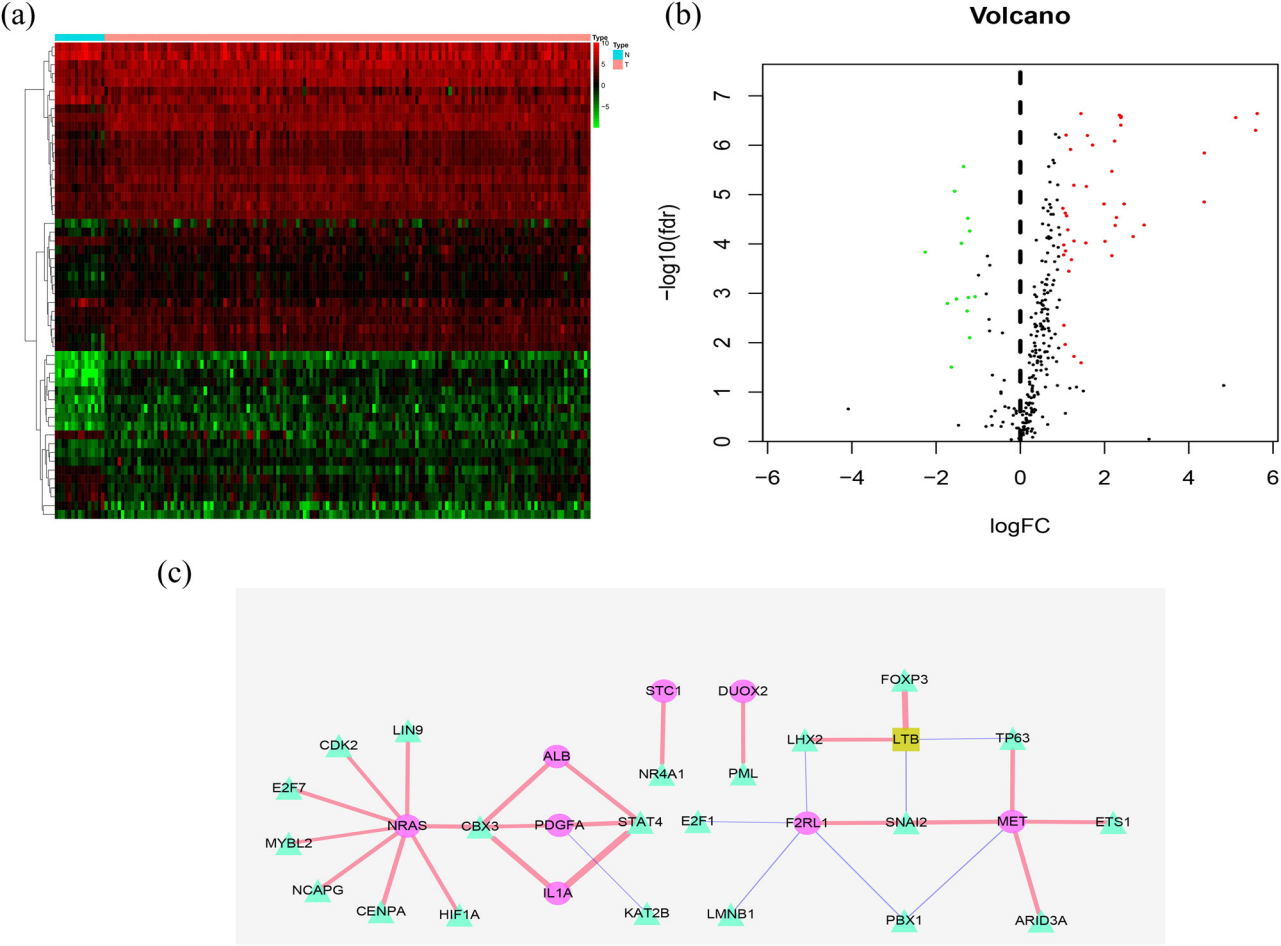
TF regulatory network: heatmap (a) and a volcano plot (b) demonstrating the differentially expressed TFs. (c) Regulatory network constructed based on clinically relevant TFs and IRGs. The negative is represented in blue and the positive in red. TF is represented as a triangle, high-risk IRGs as an ellipse, and low-risk IRGs as a rectangle. TFs, transcription factors; IRGs, immune-related genes.

We explored the potential molecular mechanisms of IRGs in TSCC samples. Among the known 318 TFs, 54 were found to have differences between the tumor and non-tumor samples, as shown in Figure 2a and b. Of these 54 TFs, 21 were involved in TSCC immunoregulation. We constructed a regulatory network with these 21 TFs and nine IRGs to demonstrate their regulatory relationships. The correlation coefficient was >0.3 (Figure 2c).

### 3.3 Identification of prognostic IRGs

We conducted univariate Cox regression analysis of the 355 screened IRGs with differential expression using the R language survival package and found 14 of them to be correlated with prognosis (dual oxidase 2 [*DUOX2*], F2R like trypsin receptor 1 [*F2RL1*], interleukin [*IL*]-*1A*, interferon regulatory factor 9 [*IRF9*], albumin [*ALB*], *NRAS*, endothelial cell specific molecule 1 [*ESM1*], gastrin [*GAST*], *IL33*, lymphotoxin beta [*LTB*], platelet derived growth factor subunit A [*PDGFA*], stanniocalcin [*STC*]-*1*, *STC2*, and *MET*). We performed a multivariate Cox regression analysis with these genes and identified seven genes for the construction of our prognostic model (*DUOX2*, *IRF9*, *GAST*, *IL33*, *LTB*, *STC1*, and *MET*) (Table 1).

**Table 1:** Univariate and multivariate Cox regression analysis evaluating predictive ability of the differentially expressed immune-related genes for OS

Gene name	Univariate cox regression analysis		Multivariate cox regression analysis	
	Hazard ratio (95% CI)	*P*-value	Hazard ratio (95% CI)	*P*-value
DUOX2	1.016(1.000–1.033)	0.043	1.032(1.013–1.052)	0.001
F2RL1	1.023(1.001–1.046)	0.040		
IL1A	1.004(1.001–1.008)	0.018		
IRF9	0.694(0.483–0.997)	0.048	0.631(0.405–0.984)	0.042
ALB	1.077(1.014–1.144)	0.016		
NRAS	1.029(1.006–1.053)	0.015		
ESM1	1.102(1.016–1.195)	0.019		
GAST	1.018(1.000–1.035)	0.046	1.019(1.003–1.036)	0.022
IL33	1.031(1.017–1.045)	<0.001	1.038(1.021–1.055)	<0.001
LTB	0.944(0.893–0.997)	0.040	0.930(0.873–0.991)	0.025
PDGFA	1.061(1.009–1.115)	0.020		
STC1	1.019(1.010–1.029)	<0.001	1.017(1.006–1.028)	0.003
STC2	1.057(1.012–1.103)	0.013		
MET	1.035(1.009–1.061)	0.008	1.037(1.010–1.65)	0.007

Notes: CI, confidence interval.

### 3.4 Construction and definition of IRGPM

**Figure 3:**
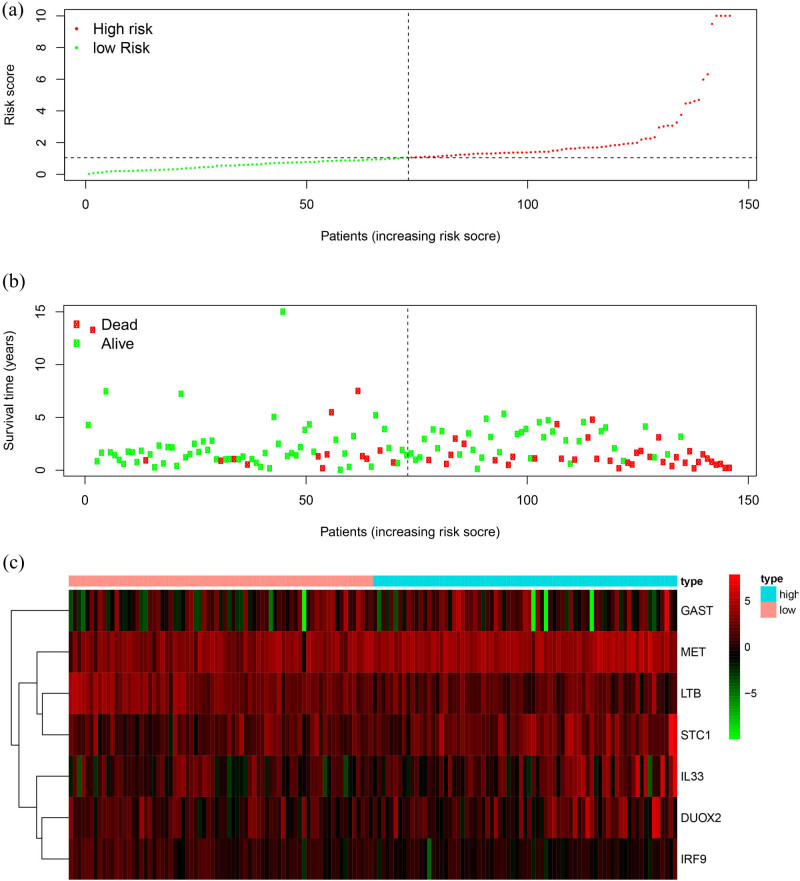
Development of the prognostic index based on IRGs: (a) rank of prognostic index and distribution of groups, (b) survival status of patients in different groups, and (c) heatmap of expression profiles of included genes. IRGs, immune-related genes.

The formula of IRGPM is as follows: XX IRGPM = (0.031880 × expression value of DUOX2) + (–0.460638 × expression value of IRF9) + (0.018953 × expression value of GAST) + (0.037026 × expression value of IL33) + (–0.072494 × expression value of LTB) + (0.0164823 × expression value of STC1) + (0.036382 × expression value of MET), of which IRF9 and LTB coefficients are negative, indicating that IRF9 and LTB were positively related to OS. Based on the results of IRGPM, we constructed a prognostic model to separate the patients with TSCC into two groups (Figure 3a–c). We used IRGPM as a variable and performed multivariate Cox regression analysis with other factors (sex, grade, stage, and histologic grade) of the TCGA dataset. The results showed that IRGPM was only correlated with OS (*P* < 0.001).

### 3.5 Validation of genes via qRT-PCR

**Figure 4:**
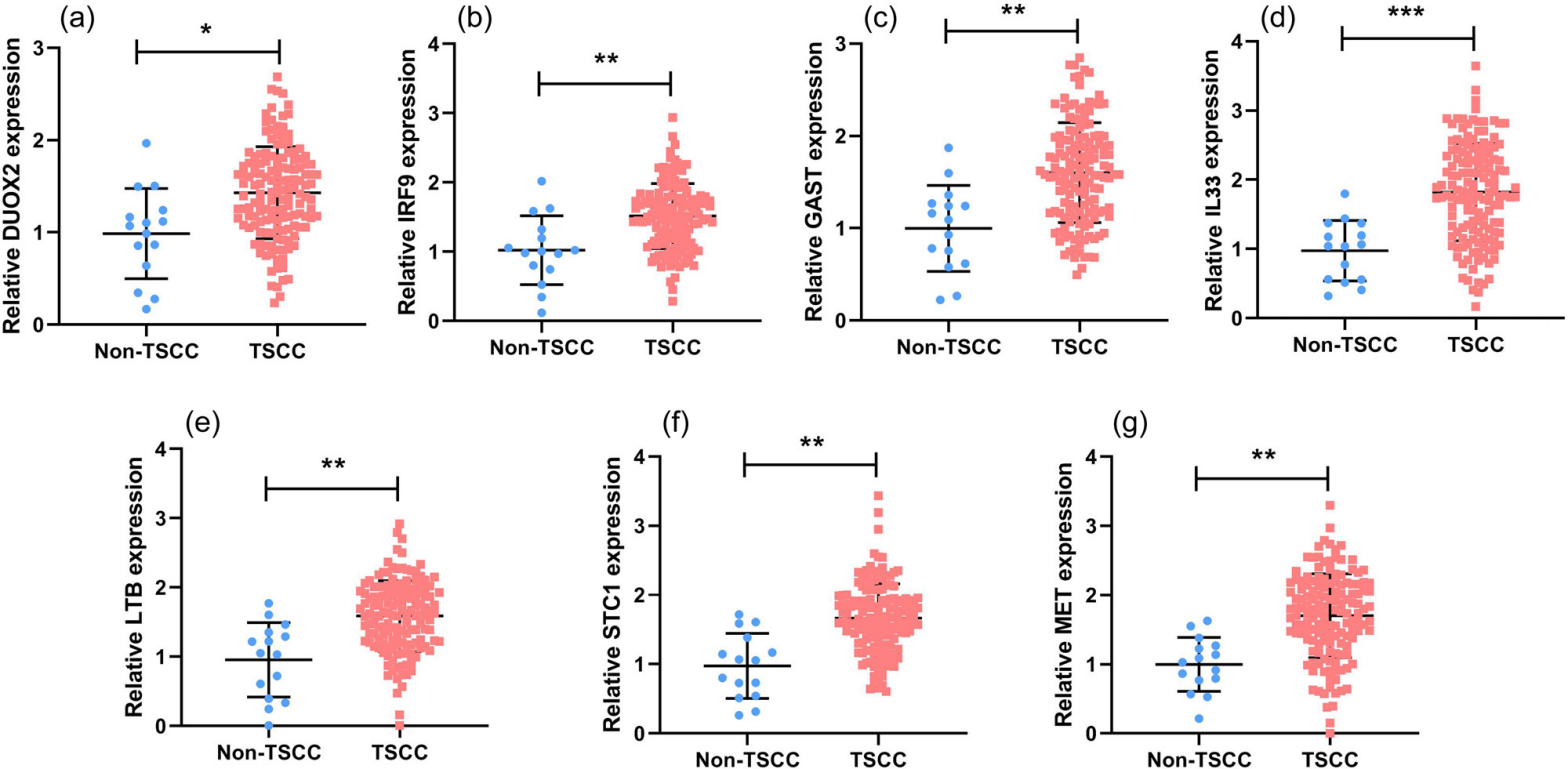
Validation of seven genes by qRT-PCR: seven genes, including (a) DUOX2, (b) IRF9, (c) GAST, (d) IL33, (e) LTB, (f) STC1, and (g) MET.

We selected seven genes related to prognosis (*DUOX2*, *IRF9*, *GAST*, *IL33*, *LTB*, *STC1*, and *MET*) for qRT-PCR analysis to verify their gene expression. As shown in Figure 4, these seven genes were significantly upregulated (*P* < 0.001), which is consistent with results of the high-throughput sequencing data.

### 3.6 Clinical outcome evaluation

**Figure 5:**
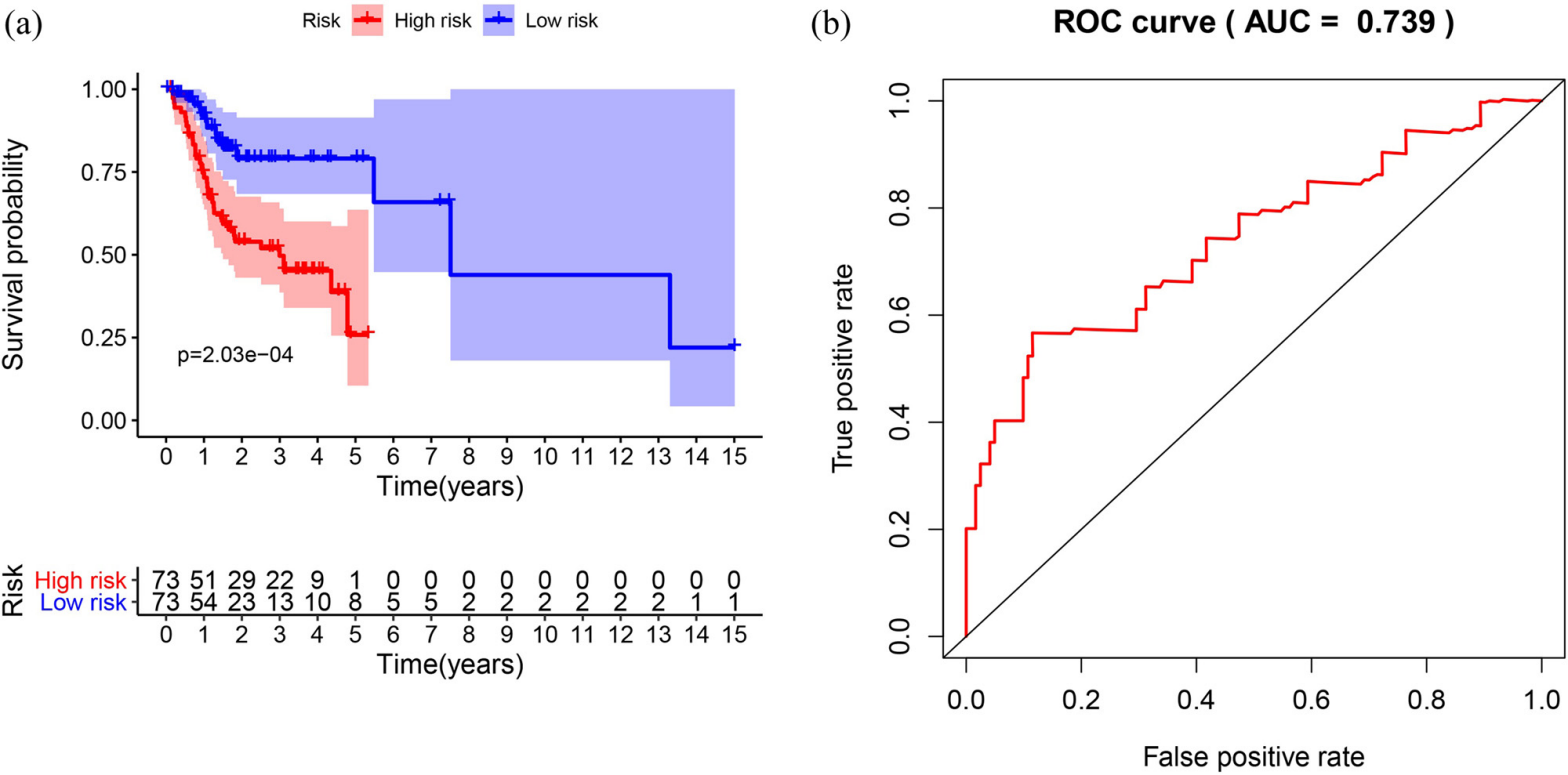
Construction and validation of the immune-related risk signature: (a) patients in the high-risk group suffered shorter OS and (b) survival-dependent ROC curve validating the prognostic value of the prognostic index. ROC, receiver operating characteristic.

According to the prognostic model, we also determined the expression levels of the prognostic genes and divided them into high- and low-risk groups and performance in terms of OS time (Figure 5a). The area under the ROC curve was 0.739 (Figure 5b), suggesting a moderate potential for the prognostic signature based on IRGs in survival monitoring. After analyzing the correlation between IRGs and clinical and pathological conditions, we found that GAST was related to grade, IRF9, LTB, and T stage (*P* < 0.05) (Table 2).

**Table 2:** The correlation between IRGs and clinical and pathological conditions

Gene name	Age		Gender		Grade		Stage		T		N	
	≤65/>65		Female/Male		G1 & 2/G3		Stage I & II/Stage III & IV		T1-2/T3-4		N0/N1-3	
	*t*	*P*	*t*	*P*	*t*	*P*	*t*	*P*	*t*	*P*	*t*	*P*
DUOX2	−1.304	0.197	1.105	0.275	−1.512	0.146	0.842	0.406	0.493	0.623	1.353	0.182
IRF9	−1.304	0.197	1.142	0.256	−0.228	0.822	1.452	0.152	2.013*	0.047	0.56	0.577
GAST	−0.582	0.565	−0.916	0.362	2.587*	0.011*	−1.937	0.055	−1.169	0.246	−1.488*	0.141*
IL33	−0.241	0.810	−0.081	0.936	−0.975	0.341	−0.49	0.625	−0.454	0.651	−0.383	0.702
LTB	0.57	0.571	−1.172	0.244	−1.742	0.094	1.663	0.104	2.74*	0.007*	−0.037	0.970
STC1	0.638	0.525	−0.937	0.351	−1.238	0.230	0.482	0.633	−0.157	0.876	0.567	0.573
MET	−0.237	0.814	0.987	0.327	−0.513	0.612	−0.386	0.701	−1.238	0.218	0.749	0.456
riskScore	0.771	0.442	−1.326	0.188	−1.485	0.153	0.315	0.755	−0.512	0.609	0.06	0.952

Notes: *t*: *t*-value of student’s *t*-test; *P*: *P*-value of student’s *t*-test; *The gene Gast was related with grade, IRF9, LTB, and T stage. *p* < 0.05.

### 3.7 Expression levels in tumor-infiltrating immune cells

**Figure 6:**
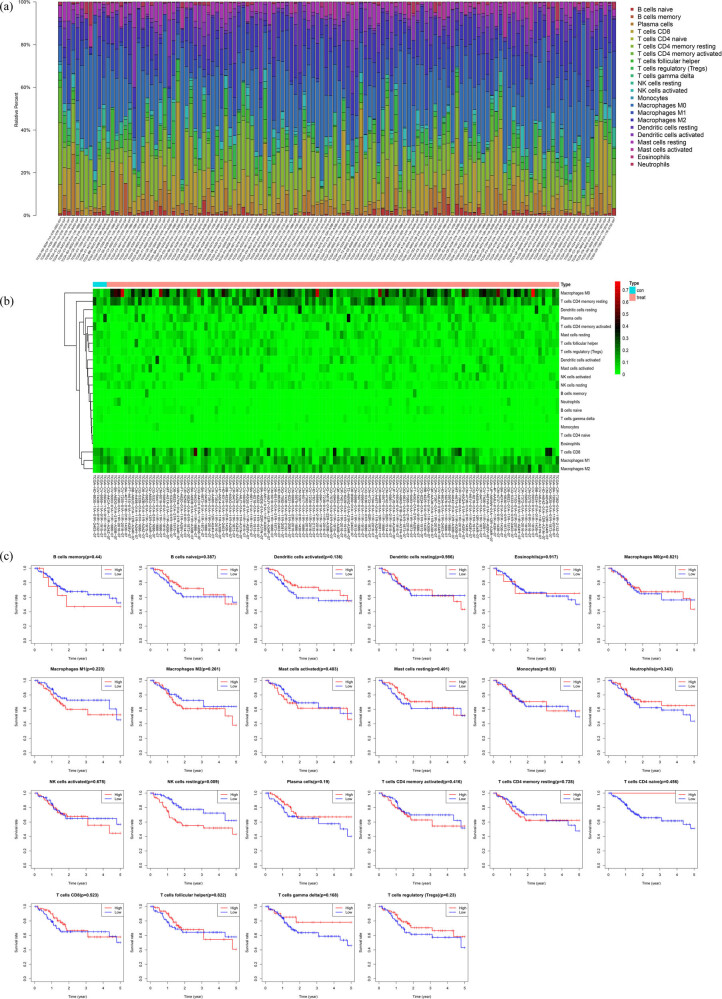
The landscape of tumor-infiltrating immune cells in TSCC: (a) the difference in tumor-infiltrating immune cells between TSCC and non-tumor tissues. (b) Heat map of the 22 immune cell proportions. The horizontal axis shows the clustering information of samples which were divided into two major clusters. (c) Association between tumor-infiltrating immune cells and 5-year survival rate in TSCC patients. Patients with a high resting NK cell population demonstrated a poor 5-year survival rate than those with a low resting NK cell population (*P* < 0.05). TSCC, tongue squamous cell carcinoma; NK cell, natural killer cell.

Using the CIBERSORT tool, we determined the relative percentage of 22 types of tumor-infiltrating immune cells in 135 samples (Figure 6a), and the cluster diagram of the 22 immune cells is shown in Figure 6b. The evaluation of the relationship between the 22 types of tumor-infiltrating immune cells and the 5-year survival in patients with TSCC showed that the resting natural killer (NK) cells and the 5-year survival rate in patients with TSCC were significantly correlated (*P* < 0.05) (Figure 6c).

## 4 Discussion

IRGs play an important role in the mechanism of tumorigenesis and have been the focus of current tumor research [17]. However, there has not yet been a systematic study involving IRGs to probe their relevance in clinical prognosis and the latent molecular mechanism in TSCC. We obtained the sample data for analysis using TCGA database and created a predictive model based on differential gene analysis. We also investigated the relative proportion of 22 types of tumor-infiltrating immune cells in tumor and non-tumor tissues along with their relationship with the survival outcome of patients with TSCC. The prognosis-related model can predict the survival outcomes of patients with TSCC. We further explored of the clinical underpinnings of TSCC by comprehensively analyzing these IRGs. Research on tumor-infiltrating immune cells provides a better understanding of their effect on the survival outcomes of patients with TSCC.

With advancements in technology, large sample data have been generated using high-throughput sequencing, which has also made gene profiling possible. Using the KEGG database for enrichment analysis, the differential expression of IRGs was found to be actively involved in a cytokine–cytokine receptor interaction pathway. The IRGs, IL33 and LTB, which constitute the prognostic model, are involved in this pathway. IL33 is a cytokine belonging to the interleukin family and plays complicated roles in the pathogenesis of autoimmune diseases and tumors. IL33 also inhibits colon cancer [18]. A portion of IL33 that is produced by lymphocytes enables c-kit + Sca + IL-7Ra, which are responsible for tumor immune surveillance and tumor tissue entry and proliferation. High levels of Th2 cytokines are produced, which differentiate into eosinophils that exhibit strong antineoplastic activity [19]. The antitumor effects of IL33 have been validated *in vitro* in mouse models [20]. However, other studies [21,22] have shown that IL33 promotes tumor development. In our study, IL33 was taken as a risk factor for TSCC, but we did not study its molecular mechanism. Amôr et al. [23] found that IL33 expression was significantly correlated with the poor prognosis of tongue cancer, suggesting that IL33-mediated malignant progression may activate the downstream signal ST2, such as nuclear factor-κB, which is a key inducer of innate immunity and inflammation and an important endogenous tumor promoter. LTB is a type II membrane protein of the tumor necrosis factor family, and was used as a protective factor in our prediction model. Although there is no direct study on whether LTB promotes or suppresses the TSCC mechanism, our findings suggest that LTB is a key molecule in mediating snail-induced cetuximab resistance in head and neck squamous cell carcinoma. Additionally, LTB itself can induce EMT [24], which is the core mechanism of tumor invasion and metastasis; loss of cell-to-cell contact via epithelial cells enables tumor migration [25]. LTB also plays a role in mediating RELB and nuclear factor-κB-2, components of the alternative nuclear factor-κB pathway; this alternative pathway promotes tumor cell migration that is an important feature of metastasis [26]. This contradicts the role of LTB in this prognostic model. Hence, further study of the mechanism underlying TSCC is essential.

We constructed a TF-involved web to reveal the regulatory roles of IRGs and found that the chromobox protein homolog 3 (CBX3) and snail family transcriptional repressor 2 (SNAI2) play vital roles. CBX3 and SNAI2 are positive regulatory factors in tumorigenesis [27]. In our study, CBX3 was found to function by upregulating NRAS, ALB, IL1A, and PDGFA levels. In an *in vitro* experimental study carried out by Zhang et al. [28], the OS rate in the high-expression TSCC patient group was significantly lower than that in the low-expression group, suggesting that CBX3 can promote TSCC cell proliferation. By knocking out CBX3, the P^21^ pathway was affected, leading to G1/S cell cycle delay. This could be a latent factor for targeted therapy in the future. SNAI2 is an EMT-promoting TF that stimulates the expression of matrix metallopeptidase 9, thereby maintaining long-term EMT promotion in head and neck squamous cell carcinoma [29]. SAN12 is positively correlated with endogenous hypoxia-inducible factor-alpha upregulation, cadherin switch, high risk of lymph node metastasis, and advanced TNM stage [30]. Mitogen-activated protein kinase (MAPK)–SNAI2 plays an important role in the metastasis of salivary adenoid cystic carcinoma. SNAI2 is the downstream target of MAPK1 (ERK2) [31]. Our network showed that SNAI2 is the main regulatory factor among the IRGs, LTBF2, RL1, and MET. This aspect is worth exploring in future studies.

NK cells, the main innate effector cells in the human body, have the natural ability to kill tumor cells and infect cells. These cells infiltrate the solid tumor site via the tumor vascular system; activated NK cells inhibit tumor growth via cytolysis of susceptible target cells and secretion of cytokines.

Resting NK cells are usually less lytic against the target cells, but they can secrete tumor necrosis factor-α and interferon-γ and kill the target cells by binding to a specific pair of receptors [32].

In this study, NK cell levels in the tumor tissues of patients with tongue cancer with a survival period of more than 5 years were compared with those of patients with a survival period of less than 5 years. The results showed that there was no difference in the number of activated NK cells between the two groups, while the inactive NK cell levels were higher in the group with a survival time of less than 5 years as compared to the group with a survival time of more than 5 years. High NK cell infiltration is correlated with better prognosis and higher survival rates in the prognosis of various solid tumors, such as lung, stomach, colorectal, and head and neck tumors [33]. However, there is no relevant literature on the quantitative assessment of the content of activated and resting NK cells in tumor tissues. Therefore, we cannot correlate this with the results of the present study.

Hasmim revealed that the tumor microenvironment consists of several cell subsets, including macrophages, myeloid inhibitory cells, T-regulatory cells, dendritic cells, cancer-related mechanocytes, and tumor cells. Their secreting factors and metabolic components have immunosuppressive effects and inhibit the activity and cytotoxicity of NK cells. NK cells can be regulated to become unresponsive in the tumor microenvironment [32]. In follow-up studies, we aim to explore the functions of different NK cell subtypes in various tumors. Our study has some limitations. First, this was a retrospective study with a limited sample size; hence, its accuracy needs to be improved. Second, further research on tumor-infiltrating immune cells and verification experiments are necessary. The role of IRGs could not reach full action, since tumors are determined not only by immune cells, but also by the intrinsic activity of tumor cells. Moreover, the overall change in dynamics was not determined and *in vitro* and *in vivo* experimental validation were not performed in this study.
